# Integrating Fermentation and Extrusion to Enhance Texture, Stability, and Nutritional Quality of Pigeon Pea (*Cajanus cajan* L.)-Based Chunk Products

**DOI:** 10.3390/foods15142514

**Published:** 2026-07-16

**Authors:** Tamara Tumasile Machinjili, José Gastão Sumila, Chawanluk Raungsri, Elsa Maria Salvador, Pavalee Chompoorat Tridtitanakiat

**Affiliations:** 1 Department of Chemical Engineering, Faculty of Engineering, Eduardo Mondlane University, Maputo 3453, Mozambique; tamarachirambo.tc@gmail.com; 2 Centre of Excellence in Agri-Food Systems and Nutrition, Eduardo Mondlane University, Maputo 1102, Mozambique; 3 Department of Community Development, Ministry of Gender, Children, Disability and Social Welfare, Lilongwe Private Bag 330, Malawi; 4 Department of Biological Sciences, Faculty of Sciences, Eduardo Mondlane University, Maputo 3453, Mozambique; sjosegastao@gmail.com (J.G.S.); elsamariasalvador@gmail.com (E.M.S.); 5 Division of Product Development Technology, Faculty of Agro-Industry, Chiang Mai University, Chiang Mai 50100, Thailand; chawanlukaung@gmail.com

**Keywords:** button mushroom, cassava starch, extrusion, fermentation, functional properties, pigeon peas, plant-based extruded snacks, proximate composition

## Abstract

The increasing global demand for affordable, nutrient-dense plant-based foods highlights the need to valorize underutilized legumes through suitable processing technologies. This study developed extruded chunk products from blends of fermented and unfermented pigeon pea (*Cajanus cajan* L.) flour, button mushroom powder, and cassava starch at ratios of 90:2.5:7.5, 90:5.0:5.0, and 90:7.5:2.5 (*w*/*w*/*w*) using a single-screw extruder. Button mushroom powder was incorporated to increase dietary fiber and mineral content due to its abundance of β-glucans and chitin, whereas cassava starch served as a texturizing and binding agent, promoting melt cohesion and expansion during extrusion. Optimal processing conditions were 13–15% feed moisture and a barrel temperature gradient of 100–140 °C. Fermentation significantly improved textural properties, increasing hardness (up to 22,802 g) and crispness (up to 55,099 g/s), likely due to protein modifications that enhanced matrix formation. In contrast, unfermented samples exhibited higher water holding capacity (264–286 g/100 g). All formulations showed low water activity (0.426–0.524), indicating good shelf stability. Protein content remained consistent (18.80–19.45%), while crude fiber ranged from 18.15 to 21.80%. These results showed that the integration of fermentation and extrusion provides an effective, low-cost approach for improving the structural, nutritional, and storage properties of pigeon pea-based products, supporting the development of nutrient-dense plant-based foods industry that are acceptable and sustainable, thereby supporting sustainable agriculture.

## 1. Introduction

Global demand for affordable, nutritionally adequate protein sources is intensifying, driven by accelerating population growth, the degradation of arable land, the persistent dominance of cereal-based dietary patterns, and rising food commodity prices in low- and middle-income countries [1,2,3]. Legumes have been repeatedly identified as strategically important crops for addressing this demand, given their capacity to fix atmospheric nitrogen, rehabilitate degraded soils, and deliver high-quality plant protein at production costs substantially lower than those of animal-based alternatives [4,5]. Among the legumes of sub-Saharan Africa, pigeon pea (*Cajanus cajan* L., family Fabaceae) occupies a position of nutritional and agronomic significance. As a drought-tolerant, short-season crop naturally adapted to the semi-arid tropics, pigeon pea is cultivated extensively across Eastern Africa including Mozambique, Malawi, Kenya, Uganda, and Tanzania, as well as across South Asia, where it constitutes a primary dietary protein source for hundreds of millions of people [6,7]. Its seed composition is notably rich in essential amino acids (lysine, methionine, and tryptophan), dietary fiber, B vitamins (riboflavin and niacin), and key minerals, including iron, phosphorus, and magnesium, a nutritional profile that positions it as a compelling functional ingredient for food product development [8,9].

Despite this nutritional and agronomic potential, pigeon pea remains chronically underutilized as a food ingredient in many of the regions where it is cultivated. In Mozambique, where production is concentrated in the provinces of Zambezia, Nampula, and Tete [10], consumption is predominantly in the form of whole cooked grain, a preparation method constrained by the crop’s characteristically long cooking time and limited recipe diversity [11]. Underutilization is compounded by marked geographic unevenness; in Malawi, for example, consumption is largely confined to the southern districts of Chiradzulu, Zomba, Blantyre, and Phalombe, with little integration into the dietary practices of other regions [11,12]. Breen C. et al. (2024) has further documented that such geographic and cultural barriers to legume adoption are systemic across sub-Saharan Africa, with consumer preference particularly among younger, urban demographics favoring convenient, shelf-stable, ready-to-eat products over traditional pulse preparations [13]. The development of innovative, value-added pigeon pea products that bypass cooking time barriers, extend shelf life, and align with evolving consumer expectations therefore represents both a market opportunity and a public health essential.

The functional and nutritional complementarity of edible mushrooms as co-ingredients in legume-based food systems has attracted increasing scientific attention [14]. Mushrooms are recognized as rich sources of non-starch polysaccharides, principally β-glucans and chitin that confer exceptional water and oil holding capacity, and which exhibit documented prebiotic, immunomodulatory, and hypocholesterolemic bioactivities [15]. Their amino acid profile, while low in methionine, complements that of legumes, and their high umami-active glutamate content enhances the flavor profile of composite food matrices [16]. The incorporation of mushroom powder into extruded snack formulations thus offers a dual opportunity to improve the functional processing behavior of the composite flour blend while simultaneously augmenting its nutritional and sensory value.

Cassava (*Manihot esculenta Crantz*) starch is one of the most widely produced and utilized starches in sub-Saharan Africa and Southeast Asia, valued for its high purity, bland flavor, and exceptional functional versatility in food processing applications [17]. As a binding and texturizing agent, cassava starch contributes superior swelling power, paste clarity, and gelatinization characteristics relative to cereal starches, making it particularly suited for thermomechanical processing operations such as extrusion [18]. Its low protein and fat content minimize competitive interactions with the legume protein fraction during melt formation, while its high amylopectin content facilitates the starch gelatinization and expansion dynamics critical to the development of an acceptable extrudate texture [19]. In the context of sub-Saharan Africa and Southeast Asia, where cassava is a staple crop cultivated across most areas, its incorporation into pigeon pea-based extruded products represents a regionally grounded, cost-effective strategy for enhancing the structural and sensory quality of the final product while supporting domestic value chain integration.

Thermomechanical extrusion processing represents one of the most versatile and scalable unit operations available for the value addition of legume-based raw materials. The simultaneous application of heat, pressure, and shear within the extruder barrel induces starch gelatinization, protein denaturation and texturization, reduction in anti-nutritional factors, and the formation of expanded, porous microstructures that underpin the textural and sensory properties of extruded snack products [20]. Extrusion has been successfully applied to a range of legume substrates, including soybean, chickpea, and lentil, producing protein-dense extrudates with commercially competitive textural profiles [21]. Fermentation of legume flours prior to extrusion introduces an additional dimension of compositional modification, partial proteolysis of storage proteins, reduction in phytic acid and trypsin inhibitor activity, and modulation of starch physiochemistry, all of which influence the rheological behavior of the flour melt and the quality attributes of the final extrudate [22]. However, the specific effects of fermentation on the extrusion behavior and physicochemical properties of pigeon pea-based composite matrices, particularly in combination with mushroom and starch co-ingredients, remain insufficiently characterized in the published literature.

In response to these identified gaps, the present study was designed to develop extruded chunk products from composite blends of fermented and unfermented pigeon pea flour, button mushroom powder, and cassava starch, in systematically varied ingredient ratios [(90:2.5:7.5, 90:5.0:5.0, and 90:7.5:2.5 (pigeon pea:mushroom:cassava)]. The study pursued three primary objectives: (i) to establish the optimal extrusion parameters, specifically barrel temperature profile and feed moisture content for the composite flour systems; (ii) to characterize and compare the physicochemical and functional properties of the resulting extruded chunks across fermented and unfermented processing conditions; and (iii) to evaluate the influence of mushroom and cassava starch inclusion levels on the quality attributes of the final product. The findings contribute to the evidence base for the valorization of pigeon pea as a high-value, shelf-stable food ingredient in sub-Saharan African food systems and beyond, with potential applicability to both smallholder processing enterprises and commercial-scale snack food manufacturing.

## 2. Materials and Methods

### 2.1. Study Design and Sites

**Figure 1:**
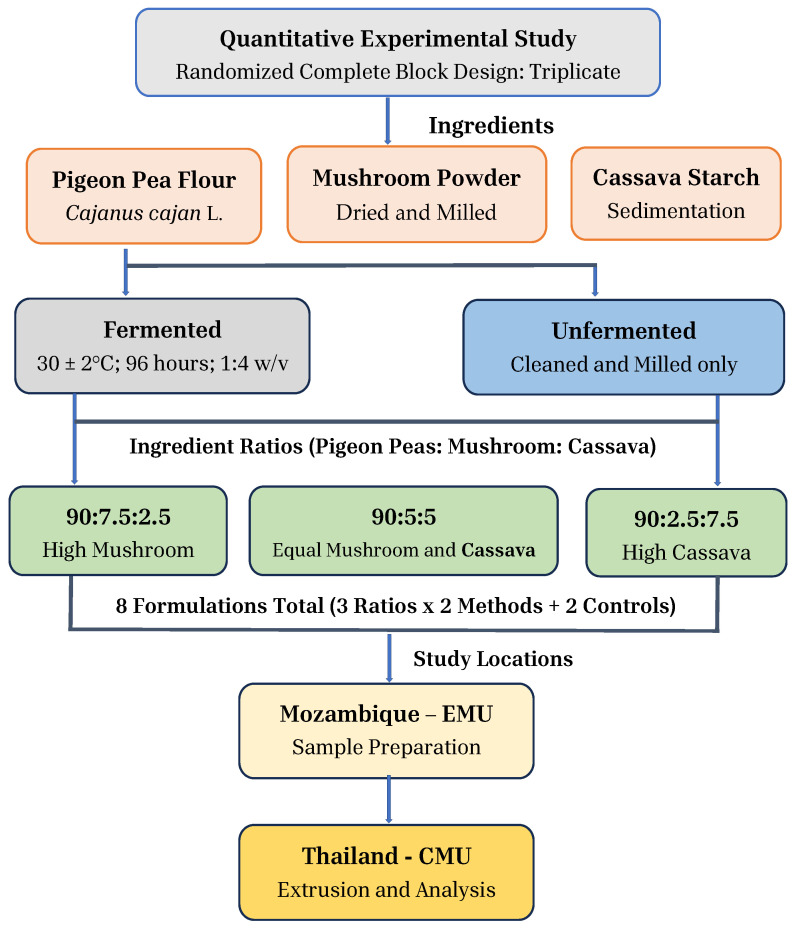
Schematic summary of the project. Note: *w*/*v*: weight to volume; EMU: Eduardo Mondlane University; CMU: Chiang Mai University.

A quantitative experimental study employing a randomized complete block design was conducted to develop and evaluate extruded chunks from composite blends of fermented and unfermented pigeon pea (*Cajanus cajan* L.) flour, mushroom powder, and cassava starch, in three ingredient ratios of 90:2.5:7.5, 90:5.0:5.0, and 90:7.5:2.5 (pigeon pea:mushroom:cassava), yielding eight formulations in total, inclusive of fermented and unfermented controls. The study was partly done in Mozambique at Eduardo Mondlane University Laboratories. Extrusion and sample analyses were done at the Faculty of Agro-Industry laboratories, Chiang Mai University, Thailand (Figure 1).

### 2.2. Sampling and Sample Size

Purposive sampling was employed to obtain samples. These samples were appropriately labeled for identification purposes. During transportation, the samples were carried in sealed Ziplock bags to maintain their integrity. Road and air transport was utilized for the transportation of samples, ensuring their safe delivery. Thirty kilograms of pigeon pea, *Cajanus cajan* L. variety, named ICEAP00557, button mushroom, and sweet cassava, were sourced from the Agricultural Research Institute of Mozambique in Maputo, Mozambique. These pigeon pea samples were manually sorted and winnowed to remove any impurities, after which they were carefully packed in Ziplock bags. The packed pigeon pea samples were stored at a temperature of 4 °C in a refrigerator and preserved for future use. The pigeon peas were cultivated during the 2023/2024 growing season.

### 2.3. Pigeon Pea Flour Preparation

Twenty kilograms (20 kg) of pigeon pea seeds were cleaned and divided into 2 equal parts of 10 kg each. Wild fermentation was conducted on one 10 kg, following the method described by Fleming et al. [23], with slight modifications. Cleaned pigeon peas were completely submerged in a 10% brine solution prepared by dissolving 100 g of locally sourced non-iodized salt in 1 L of chlorine-free water. The mixture was placed in a plastic bucket, covered with a cotton cloth, and allowed to ferment in a dark room at ambient temperature for 96 h. After fermentation, the samples were sun dried for 48 h. Both the fermented and unfermented samples were then milled into a fine powder using a laboratory hammer mill (Model LM 3100, PerkinElmer, Stockholm, Sweden) to fine flour (1 mm mesh sieve). The samples were kept in Ziplock plastic bags until analysis.

### 2.4. Preparation of Button Mushroom Powder

The mushrooms were washed with clean water to remove dirt, sand, and other undesirable materials before use. The fresh, clean mushroom samples were sun dried till up to 11% moisture content, then oven dried (model no. DSO-500D/DSO-500DF, GRANDE Automatic Test Equipment Limited, Dongguan, China) at 60 °C and a relative air humidity of 75%. Drying was finished when a constant moisture content of 11% was attained. The dried mushroom slices were cooled and milled in a laboratory hammer mill (Model LM 3100, PerkinElmer, Stockholm, Sweden) to fine flour (1 mm mesh sieve) and packaged in Ziplock plastic bags until analysis.

### 2.5. Preparation of Cassava Starch

Starch was extracted by the sedimentation method [18]. The cassava tubers were sorted out, weighed, and washed with clean water. Tubers were peeled, washed, cleaned to remove all dirt, and cut into chunks of about 0.5–1 cm thick. The chunks were milled using a blender with about 200 g of water to blend each 100 g sample smoothly. The slurry was filtered through a clean cheese cloth. The solids retained by the cloth were washed with more water and filtered through the cheese cloth. The washing process was repeated five times until there was little or no starch in the filtrate. Starch in the filtrate was allowed to sediment overnight and the liquid was decanted and discarded. The starch was dried in a solar drier until dried and then weighed. The samples were packaged in Ziplock plastic bags until analysis.

### 2.6. Extrusion of the Pigeon Pea Chunks

A single-screw extruder (Do-Corder C3 DN19/20, Brabender, Duisburg, Germany), operated at 15 rpm of feed rate and 185, 217, and 241 rpm of screw speed, was used for development of the extruded products. The barrel diameter and length-to-diameter (L:D) ratio were 37 mm and 27:1, respectively, with the screw configuration standardized for processing flour-based products. The barrel was provided with two electric band heaters and two water cooling jackets. A temperature sensor was fitted on the front die plate, which was connected to temperature control system on the panel board. The screw profile was made up of conveying self-wiping elements, except for a section consisting of short reverse and forwarding elements, to improve mixing and apply shear to the material being extruded, while restricting flow and building up pressure. The exit diameter of the circular die was 3 mm. A volumetric feeder was used for feeding the dry mixture to the extruder. A trial on the development of chunks was done to find the recommended temperature and moisture content for the ingredients. The flour mixtures were set on three different moisture contents, which were 11%, 13%, and 15%, to determine the right moisture content for processing. The temperature was set at different degrees Celsius in three zones of the barrel, which were 100 °C, 130 °C, 150 °C, and 170 °C.

### 2.7. Functional Properties of Pigeon Pea Flour

#### 2.7.1. Bulk Density

Bulk density (BD) was determined following the method described by Njintang et al. (2001), with modifications. Briefly, 100 g of pigeon pea flour was transferred into a 100 mL graduated cylinder containing 50 mL of deionized water at 25 ± 2 °C. The sample was allowed to hydrate for 10 min to reach equilibrium, and the displaced water volume was recorded. BD was calculated as the ratio of sample mass (g) to the volume (mL) of displaced water [24].

$$\mathrm{BD}\;(g/\mathrm{mL})=\frac{\mathrm{mass}\;(g)}{\mathrm{volume}\;\mathrm{of}\;\mathrm{sample}\;(\mathrm{mL})}$$

All measurements were conducted in triplicate, and results are expressed as mean ± standard deviation.

#### 2.7.2. Swelling Index

The swelling index (SI) was determined according to the procedure of Adebowale et al. (2005). Briefly, 2.5 g of flour was suspended in 25 mL of distilled water in a pre-weighed centrifuge tube and incubated at 25 °C for 30 min with occasional agitation. The suspension was then centrifuged at 3000 rpm for 15 min, and the supernatant was carefully decanted. The weights before ($m_{o}$) and after ($m_{s}$) immersion were recorded. The SI was calculated using the following equation [25]:

$$\mathrm{SI}\;(\%)=\left(\frac{m_{s}}{m_{o}}\right)\times 100$$

#### 2.7.3. Solubility

Solubility was determined based on the method of Onwuka (2005). A 2 g sample was dispersed in 40 mL of distilled water at 25 °C under continuous stirring for 30 min. The suspension was centrifuged at 3500 rpm for 20 min. A 10 mL aliquot of the supernatant was transferred into a pre-weighed evaporating dish and dried at 105 °C to constant weight. Solubility was calculated as follows [26]:

$$\mathrm{Solubility}\;(\%)=\frac{W_{2}\times V_{\mathrm{total}}}{V_{\mathrm{aliquot}}\times W_{1}}\times 100$$

where $W_{1}$ is the initial sample mass (g), $W_{2}$ is the dried supernatant residue mass (g), $V_{\mathrm{total}}$ is the total water volume (mL), and $V_{\mathrm{aliquot}}$ is the supernatant volume taken for drying (mL). All measurements were performed in triplicate.

#### 2.7.4. Water Holding Capacity and Oil Absorption Capacity

Water holding capacity (WHC) was assessed using the centrifugation method of Beuchat (1977) with some modifications. Briefly, 3 g of flour was mixed with 10 mL of distilled water in a pre-weighed centrifuge tube, stirred for 1 min, and left to stand for 30 min at 25 °C. The mixture was centrifuged at 3000 rpm for 20 min. The supernatant was decanted, and WHC was calculated as grams of water retained per gram of sample [27].

$$\mathrm{WHC}\;(\%)=\frac{\mathrm{weight}\;\mathrm{of}\;\mathrm{bottles}\;\mathrm{after}\;\mathrm{decanting}-\mathrm{weight}\;\mathrm{of}\;\mathrm{dry}\;\mathrm{tube}-\mathrm{total}\;\mathrm{flour}\;\mathrm{weight}}{\mathrm{weight}\;\mathrm{of}\;\mathrm{sample}\;(g)}$$

Oil absorption capacity (OAC) was determined according to the procedure described by Lin et al. (1974) with some modifications. A 3 g sample was mixed with 5.0 mL of soybean oil, stirred, and centrifuged at 3000 rpm for 15 min. The free oil was decanted, and the pellet was drained for 30 min. OAC was expressed as grams of oil absorbed per gram of sample. Oil holding capacity (OHC) for extruded chunks was calculated using the formula [28]:

$$\mathrm{OHC}\;(\%)=\left(\frac{W_{2}}{W_{1}}\right)\times 100$$

where $W_{1}$ and $W_{2}$ are the sample weights before and after centrifugation, respectively.

#### 2.7.5. Water Activity

Water activity ($a_{w}$) was measured using a Decagon AquaLab Pre water activity analyzer (Decagon Devices, Inc., Pullman, WA, USA). Approximately 5 mg of flour was placed in the sample holder, and measurements were performed in triplicates at 25 °C immediately after drying [29].

### 2.8. Physicochemical Properties of Pigeon Pea Chunks

#### 2.8.1. Moisture Content

Moisture content was determined using a Precisa XM-60 moisture analyzer (Precisa Gravimetrics AG, Dietikon, Switzerland) according to the manufacturer’s instructions. All measurements were done in triplicates. Results were expressed as a percentage on a wet weight basis [30].

#### 2.8.2. Protein Content

Total nitrogen content was determined by the Dumas combustion method, as described by [31]. Homogenized samples (10 mg each) were wrapped in nitrogen-free tin foil, pressed, and combusted at 990 °C for 300 s under pure oxygen in a combustion tube (reduction tube temperature: 650 °C). The limit of quantification was 0.1 g/100 g. Crude protein content was calculated using a conversion factor of 6.25 [32]:

Protein (%)=Total Nitrogen (%)×6.25

All analyses were performed in triplicate.

#### 2.8.3. Crude Fat Content

Crude fat was determined using an automated Soxhlet extraction unit (Soxhlet TM 8000, Foss, Hoganas, Sweden), following AOAC Official Method 991.36 [33]. Petroleum ether was used as the solvent, and fat content was expressed as a percentage (*w*/*w*).

#### 2.8.4. Crude Fiber Content

Crude fiber was analyzed according to AOAC Official Method 962.09 [34]. One gram of defatted sample was boiled in 200 mL of 1.25% H_2_SO_4_ at 100 °C for 30 min with stirring. The mixture was filtered and washed with hot distilled water until neutral. The residue was then boiled in 200 mL of 1.25% NaOH at 100 °C for 30 min, filtered, and washed with 15 mL of 96% ethanol followed by hot distilled water. The final residue was dried at 100 °C to constant weight. Crude fiber was calculated as weight loss after incineration.

$$\mathrm{Crude}\;\mathrm{fiber}\;\%=\frac{b-a}{\mathrm{Weight}\;\mathrm{of}\;\mathrm{sample}}\times 100$$

where b is the weight of the crucible and dried residual before ashing and a is the weight of the crucible and ash after ignition.

#### 2.8.5. Carbohydrate Content (By Difference)

Total carbohydrate content was determined by difference using the following equation as described by Food and Agriculture Organization (2003) [35]:

Carbohydrate (%)=100−(%moisture+%protein+%fat+%crudefiber+%ash)

#### 2.8.6. Ash Content

Ash content was measured according to AOAC Official Method 923.03 [36]. Two grams of sample were ashed in a pre-ignited porcelain crucible in a muffle furnace at 550 °C for 6 h until a light gray ash was obtained. The crucible was cooled in a desiccator and weighed to constant mass. Ash content was expressed as a percentage (*w*/*w*) on a dry basis.

#### 2.8.7. pH Measurement

The pH of the flour was determined according to the method of AOAC Official Method 943.02 [37]. Ten grams of sample were dissolved in 100 mL of distilled water and shaken thoroughly to obtain a homogeneous mixture. The pH was measured using a calibrated pH meter (Model 3520, Bibby Scientific Ltd., Dumow, UK), standardized with pH 4.0 and 7.0 buffer solutions.

#### 2.8.8. Texture Measurement

Texture analysis was performed using a Stable Micro Systems TA.XT plus extended height texture analyzer (TA-XT plus, Stable Micro Systems Ltd., Surrey, UK) following the procedure of Bourne (2002). Crispness was evaluated using a puncture probe, recording the number of force peaks and maximum fracture force from 10 replicates per sample. Hardness was determined by texture profile analysis (TPA) using a 35 mm cylindrical probe (P/35) compressed to 40% of the sample height at a rate of 1.0 mm/s. Test conditions were: pre-test speed 1 mm/s, test speed 2 mm/s, post-test speed 10 mm/s, deformation 40%, and auto trigger force 5 g. Hardness was expressed as the maximum peak force (N) during the first compression cycle, representing the resistance to breakage [38].

### 2.9. Statistical Analysis

All determinations of functional and physicochemical properties were conducted in triplicate. Determination of texture (hardness and crispness) using the standardized method of the texture analyzer was done in 10 replicates. Data were analyzed using a generalized linear model with two-way analysis of variance (ANOVA), considering processing method (fermented and unfermented) and formulation ratios as fixed factors. Statistical analysis was performed using IBM SPSS Statistics, version 29.0 (Armonk, NY, USA). When significant differences were detected (*p* < 0.05), means were separated using the least significant difference (LSD) test.

## 3. Results

### 3.1. Functional Properties of Flours

**Figure 2:**
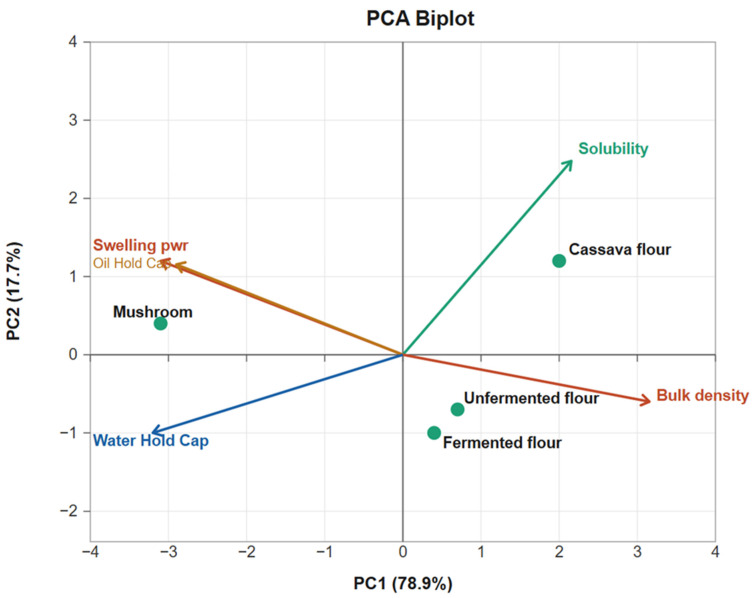
Principal Component Analysis (PCA) biplot illustrating the functional property relationships among the four raw flour ingredients used in extrudate formulation: fermented pigeon pea flour (FPP), unfermented pigeon pea flour (UPP), button mushroom powder (BMP), and cassava starch (CS). Vectors represent the five functional property variables (swelling power, solubility, water holding capacity, oil holding capacity, and bulk density); the length and direction of each vector indicate the magnitude and pattern of variation among samples. Samples positioned in the direction of a vector have higher values for the corresponding property. The biplot was generated from standardized data using IBM SPSS Statistics v29.0.

The results indicate significant differences (*p* < 0.05) among samples for all functional properties. Mushroom exhibited the highest swelling power, water holding, and oil holding capacities, but the lowest bulk density, suggesting superior hydration and lipid-binding potential. Fermented pigeon pea flour showed enhanced water holding capacity compared to unfermented and cassava flours, indicating improved functional modification. Cassava flour had the highest solubility, while unfermented pigeon pea flour generally showed lower functional performance. These variations reflect the influence of fermentation and raw material composition on physicochemical properties (Table 1, Figure 2).

**Figure 3:**
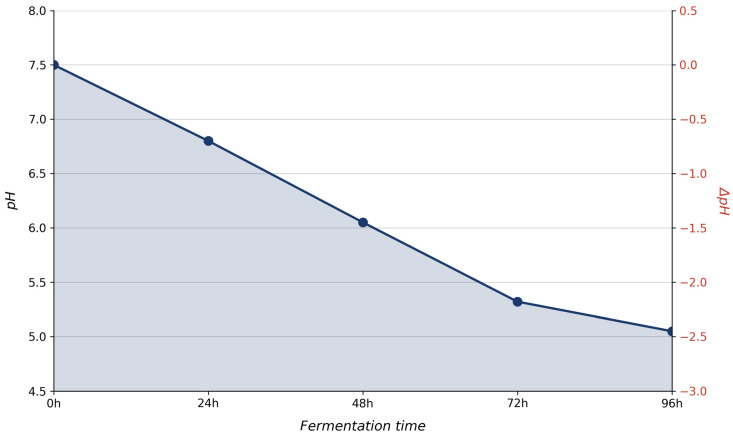
pH profile during 96 h wild (spontaneous) fermentation of pigeon pea (*Cajanus cajan* L.) seeds in 10% brine solution at ambient temperature. Mean pH values (n = 3) were recorded at 0, 24, 48, 72, and 96 h. The monotonic decline from pH 7.51 (0 h) to pH 5.06 (96 h) reflects progressive lactic acid bacterial acidification, with peak acidification rate occurring between 24 and 48 h and a decelerating phase from 48 to 96 h consistent with substrate depletion and product inhibition.

The evolution of pH throughout the fermentation period was monitored as a primary indicator of acidogenic metabolic activity. As illustrated in Figure 3, the initial pH of 7.51 declined progressively and consistently over the 96 h fermentation period, reaching a terminal value of approximately 5.06, representing a total acidification of ΔpH = −2.45 units.

### 3.2. Proximate Composition of Flours

The proximate composition of the four flour samples varied considerably across most parameters. Cassava starch recorded the highest carbohydrate content (85.75%) but the lowest protein (1.30%), crude fat (0.81%), ash (0.79%), and crude fiber (0.26%). Mushroom flour had the highest ash content (13.11%), while fermented and unfermented pigeon pea flour exhibited comparable protein levels (18.87% and 19.17%, respectively), with no significant difference observed (*p* = 0.355). Moisture content differed significantly among samples (*p* = 0.002), with all flour samples recording values between 10.96% and 11.09% (Table 2).

### 3.3. Recommended Parameters for Developing Chunks

**Figure 4:**
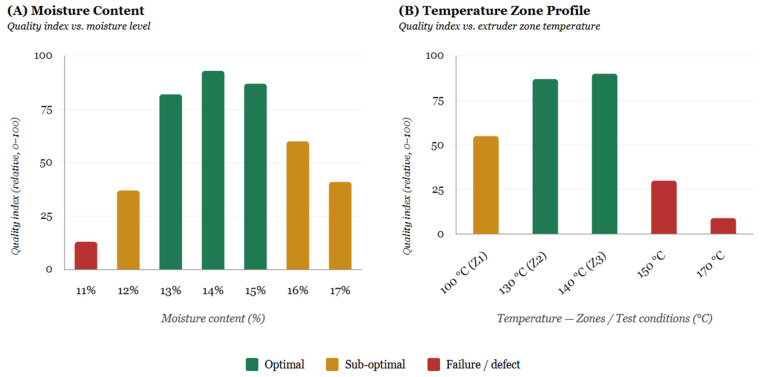
Parameters for chunk development. (**A**) Effect of feed moisture content and (**B**) barrel temperature profile on the relative quality index of extrudates produced from the composite pigeon pea:mushroom:cassava starch blends. The quality index shown in Figure 4 was a composite score used to assess the suitability of extrusion conditions based on the physical quality of the resulting extrudates. The index was expressed on a 0–100 scale, with higher scores indicating superior product quality. Scores were assigned by evaluating expansion characteristics, texture (hardness and crispness), structural integrity, and the absence of processing defects such as collapse or burning. Products were classified as optimal (≥75), sub-optimal (25–74), or defective (<25). The quality index served as a comparative tool for identifying extrusion moisture and temperature conditions that produced the most desirable product attributes.

The optimal moisture content for extrusion was determined to be in the range of 13–15%, which facilitated adequate flow and expansion within the extruder. When the moisture level was reduced to 11%, the extrudates exhibited excessive consistency, resulting in poor conveyance through the barrel and subsequent processing difficulties. Temperature profiling across the extruder zones was also critical for achieving desirable product quality. Specifically, a gradient of 100 °C in Zone 1, followed by 130 °C and 140 °C in Zones 2 and 3, respectively, ensured proper starch gelatinization and texturization. However, at elevated temperatures of 150 °C and 170 °C, thermal degradation occurred, leading to charring and unacceptable product quality (Figure 4).

### 3.4. Physicochemical and Functional Properties of Extruded Chunks

**Figure 5:**
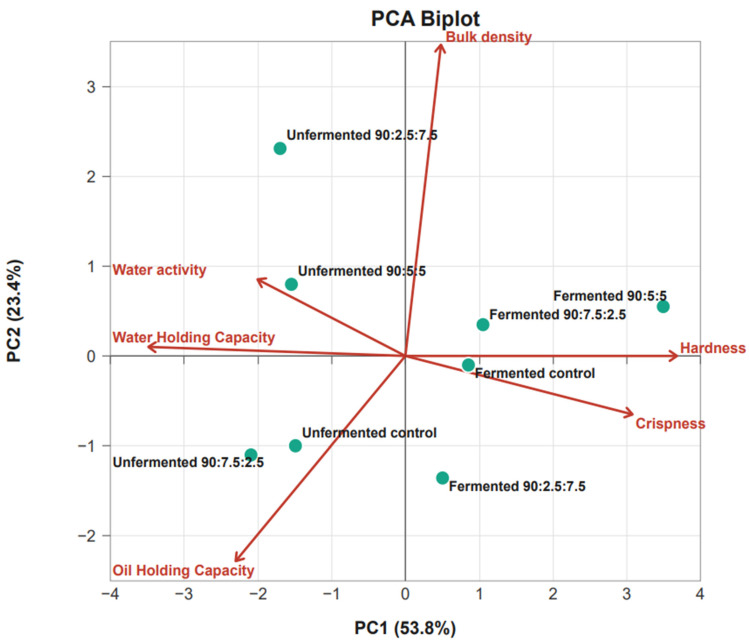
Principal Component Analysis (PCA) biplot of the physicochemical and functional properties of the eight extruded chunk formulations: four fermented (F-90:7.5:2.5, F-90:5:5, F-90:2.5:7.5, fermented control) and four unfermented (UF-90:7.5:2.5, UF-90:5:5, UF-90:2.5:7.5, unfermented control). Variables plotted include bulk density, water holding capacity (WHC), oil holding capacity (OHC), water activity, hardness, and crispness. The biplot illustrates the clear separation between fermented and unfermented formulations along the principal components, driven primarily by hardness and crispness in fermented samples, and WHC in unfermented samples.

The results show significant differences (*p* < 0.05) in water holding capacity, water activity, hardness, and crispness, while bulk density and oil holding capacity were not significantly affected. Unfermented samples exhibited higher water holding capacity, indicating greater hydration potential. Fermented formulations showed markedly higher hardness and crispness, particularly at the 90:5:5 ratio, suggesting enhanced structural integrity and expansion during extrusion. Lower water activity in fermented samples implies improved shelf stability (Table 3, Figure 5).

### 3.5. Proximate Composition of Extruded Chunks

The proximate composition shows significant differences (*p* < 0.05) in moisture, crude fat, crude fiber, ash, and carbohydrates, while protein content remained statistically similar across samples. Fermented products generally exhibited lower crude fiber but higher fat variability, particularly in the control. Unfermented samples showed higher fiber content, reflecting limited biochemical modification. Carbohydrate content was highest in fermented 90:2.5:7.5, suggesting compositional shifts during fermentation. Overall, fermentation influenced nutrient distribution, especially fat and carbohydrate fractions, without significantly altering protein levels (Table 4).

## 4. Discussion

The results of the present study demonstrate that the development of extruded chunk products from composite blends of fermented or unfermented pigeon pea flour, button mushroom powder, and cassava starch is technically feasible under the optimized processing conditions identified, and that the resulting products exhibit nutritional and functional profiles that position them competitively within the landscape of plant-based snack foods. The application of brine fermentation to pigeon pea prior to extrusion constitutes a low-cost, scalable intervention that significantly modifies the textural and stability attributes of the extruded product without damaging the protein content as observed in similar studies [22]. Extrusion of pigeon pea, mushroom, and cassava starch blends produced shelf-stable chunks with protein contents of 18.8–19.45% and crude fiber of 18–22%, preserved across all formulations irrespective of processing method (*p* = 0.424). Fermentation significantly increased extrudate hardness and crispness through partial proteolysis of the protein–starch melt network [21], while unfermented chunks exhibited superior water holding capacity attributable to a more porous post-extrusion microstructure. Water activity values (0.426–0.524) fell below the 0.60 spoilage threshold across all samples, confirming microbiological shelf stability. The optimal extrusion window was established at 13–15% feed moisture and 100–140 °C barrel temperature.

### 4.1. pH Profile for Wild Fermentation of Pigeon Peas and Changes over Time

The data demonstrate a clear and progressive acidification of the fermentation system, with pH decreasing from near-neutral (7.51 at 0 h) to mildly acidic (5.06 at 96 h), representing a total reduction of 2.45 units. This monotonic decline is consistent with the metabolic activity of lactic acid bacteria (LAB) and other indigenous acidogenic microorganisms typical of spontaneous fermentation. The initial pH reflects the intrinsic buffering capacity of pigeon pea seeds, largely attributable to proteins, phosphate groups, and soluble minerals, which is characteristic of protein-rich legumes [39].

The progressive decline in pH from 7.51 to 5.06 over 96 h reflects sustained acidogenic fermentation activity, consistent with the metabolic production of organic acids—predominantly lactic and acetic acids, as primary end-products of carbohydrate catabolism [40]. The near-linear acidification observed during the initial 48 h corroborates findings in analogous fermentation systems, wherein active microbial proliferation correlates directly with accelerated proton accumulation in the fermentation medium, a pattern well described by growth-associated kinetic models such as the Luedeking–Piret equation [41]. The subsequent attenuation of the acidification rate beyond 48 h is characteristic of the deceleration phase, wherein diminishing substrate availability and accumulating organic acid concentrations progressively constrain microbial metabolic output through combined substrate depletion and product inhibition mechanisms [42]. The terminal stabilization of pH near 5.0 is particularly noteworthy, as values within this range have been widely reported to exert selective pressure on microbial community composition, favoring acid-tolerant taxa, notably lactic acid bacteria, while effectively suppressing competing spoilage and pathogenic microorganisms, a phenomenon of considerable relevance to both fermentation efficiency and product microbiological safety [39,43].

### 4.2. Functional Properties of Raw Flour Ingredients

The functional properties of the raw flour ingredients played a big role in the processing behavior of the composite blends during extrusion and for the textural quality of the resultant extrudates. Mushroom powder exhibited the highest swelling power (516.4%), water holding capacity (308.5%), and oil holding capacity (244.69%) among all tested ingredients, but the lowest bulk density (0.40 g/mL), reflecting the hygroscopic, porous microstructure characteristic of dried fungal tissue [44]. The extraordinary swelling power of mushroom powder is attributable to the high concentration of β-glucans and chitin, which possess an exceptional capacity for hydration and volumetric expansion upon contact with water. Comparable swelling power values for oyster mushroom (*Pleurotus ostreatus*) powder have been reported in the range of 450–560%, consistent with the present observation [45].

Fermented pigeon pea flour demonstrated significantly enhanced water holding capacity (272.27%) relative to unfermented pigeon pea flour (194.13%). Fermentation-induced partial proteolysis increases the number of exposed hydrophilic amino acid residues and disrupts compact protein quaternary structures, yielding protein fragments with greater surface hydration capacity [22]. Badia-Olmos et al. (2024) reported similar enhancements in water absorption indices of fermented cowpea and lentil flours, while Verni et al. (2022) attributed increased water holding capacity of fermented legumes to the unfolding of legumin and vicilin-type globulins under acidic pH conditions generated during fermentation [46,47]. The functional improvement in fermented pigeon pea is directly relevant to textural characteristics.

Cassava starch exhibited the highest solubility (161.1%) among all tested flours, a characteristic attributable to its high amylopectin content and the structural fragility of cassava starch granules under aqueous conditions [48]. Research has shown that cassava starch has exceptionally high water solubility relative to maize and potato starches, a property linked to the low degree of granule crystallinity and the highly branched amylopectin architecture [49]. In the context of the extruded product, the high solubility of cassava starch contributes positively to starch melt formation and the development of a homogeneous, cohesive extrudate matrix [50]. The low bulk density of mushroom powder (0.40 g/mL) compared to the pigeon pea flour (0.63 g/mL) and cassava starch (0.67 g/mL) indicates a more porous, aerated particle structure that may influence the packing behavior of the composite flour blend during feeder conveyance and subsequently, affect specific mechanical energy inputs during extrusion [51].

### 4.3. Proximate Composition of Raw Flour Ingredients

The proximate composition of the four ingredients characterized in the present study revealed distinct nutritional profiles that collectively rationalize the formulation strategy adopted. Pigeon pea flour, in both its fermented and unfermented forms, exhibited protein contents of 18.87% and 19.17% (dry weight basis), respectively, with no statistically significant difference between the two conditions (*p* = 0.355). These values are consistent with the protein concentration range of 18–25% reported for various pigeon pea genotypes in the literature. Others have documented protein contents of 18.7–21.7% for pigeon pea accessions, while others have similarly reported values of approximately 20.0% for mature dry pigeon pea seeds [6,9,52,53]. The comparability of protein content between fermented and unfermented samples in the present study aligns with other research findings, which demonstrated that solid-state and brine fermentation of pea and navy bean did not significantly reduce total protein content, though they may induce redistribution of nitrogen fractions through partial proteolysis of storage proteins into lower-molecular-weight peptides and free amino acids [54].

Fermented pigeon pea flour exhibited a numerically higher crude fat content (7.09%) compared to its unfermented counterpart (5.70%), a difference that attained statistical significance (*p* < 0.001). This observation is somewhat counterintuitive and may be attributable to fermentation-induced hydrolysis of cell wall polysaccharides, which could liberate previously physically entrapped lipid fractions and render them extractable [55]. A comparable phenomenon was reported in fermented bambara beans and soybean, where fermentation increased ether-extractable fat due to enhanced cellular accessibility [56]. The notably elevated crude fat content of unfermented pigeon pea (5.70%) relative to the cassava starch (0.81%) reflects the inherently lipid-rich cotyledon structure of legume seeds [57].

Crude fiber content was substantially higher in pigeon pea flours (fermented: 20.82%; unfermented: 21.86%) and mushroom powder (20.52%) relative to cassava starch (0.26%). The marginally lower fiber content observed in fermented pigeon pea compared to its unfermented counterpart is consistent with partial microbial degradation of structural cell wall polysaccharides, particularly hemicellulose and pectin, during the 96 h brine fermentation [55]. Lactic acid bacteria and other fermentative microorganisms associated with spontaneous legume fermentation produce cell wall-degrading enzymes that progressively reduce dietary fiber content [58]. The high dietary fiber content of mushroom powder (20.52%), predominantly comprising chitin and β-glucans, is in agreement with values reported for *Astraeus odoratus* and *Agaricus bisporus* mushrooms [59,60], and represents a functional advantage for the composite extrudate, as β-glucan-type non-starch polysaccharides have documented prebiotic and cholesterol-lowering bioactivities [61].

Mushroom powder recorded the highest ash content (13.11%) among all ingredients, significantly exceeding those of pigeon pea flours (4.46–4.80%) and cassava starch (0.79%). This exceptionally elevated mineral content in mushroom powder is a well-documented phenomenon attributable to the high accumulation of potassium, phosphorus, selenium, and trace elements from the growth substrate [62]. The inclusion of mushroom powder in the composite formulation therefore offers a strategy for mineral fortification of the extruded product, complementing the iron and phosphorus contributed by the pigeon pea fraction. Cassava starch recorded the highest carbohydrate content (85.75%), which is consistent with its recognized role as a predominantly starchy substrate with minimal protein, fat, and fiber fractions [19]. This composition positions cassava starch as the primary contributor to starch gelatinization dynamics and post-extrusion expansion characteristics within the composite system.

### 4.4. Optimization of Extrusion Processing Parameters

The optimization of extrusion parameters is central to achieving acceptable physicochemical and textural properties in legume-based extrudates. In the present study, the optimal feed moisture content for the composite pigeon pea:mushroom:cassava starch blends were determined to lie within the range of 13–15%, while a barrel temperature gradient of 100 °C (Zone 1), 130 °C (Zone 2), and 140 °C (Zone 3) yielded products of acceptable quality without thermal degradation. These findings are broadly consistent with the extrusion literature for comparable legume-based composite systems [21,63].

The inability to achieve acceptable extrudate quality at a feed moisture content of 11% is contributed mechanistically; at sub-optimal moisture levels, the composite melt within the extruder barrel attains excessively high viscosity, impeding conveyance and generating torque overload conditions that reduce residence time homogeneity and extrudate uniformity [64]. Similarly, others have observed poor extrusion behavior for composite blends at feed moisture contents below 12%, attributing this to incomplete plasticization of the protein–starch matrix and excessive frictional heating and reported optimal feed moisture contents of 14–16% for soy protein-based extrudates, further corroborating the moisture range identified in the present study [65].

The upper temperature limit of 140 °C in Zone 3, beyond which charring and unacceptable thermal degradation occurred, reflects the thermolabile nature of the pigeon pea protein fraction and the susceptibility of the composite melt to Maillard browning and pyrolysis reactions at elevated temperatures [66]. Others have documented significant darkening and reduction in expansion ratio for legume-based extrudates processed above 150 °C, attributing this to excessive starch dextrinization and irreversible protein cross-linking [67]. Conversely, the progressive temperature gradient from 100 to 140 °C established in the present study ensured sequential moisture removal, starch gelatinization initiation in the intermediate zones, and complete gelatinization with controlled melt expansion at the die exit, a profile consistent with best practices for legume–starch composite extrusion [68].

### 4.5. Physicochemical and Functional Properties of Extruded Chunks

Following extrusion processing, the physicochemical and functional properties of the extruded chunks were significantly influenced by both the fermentation status of the pigeon pea flour and the relative proportions of mushroom powder and cassava starch in the composite blend. The absence of significant differences in bulk density across all extruded formulations (*p* = 0.083) suggests that the structural expansion of the extrudate, which is the primary determinant of bulk density, was similarly achieved across all formulations under the optimized processing conditions. This may reflect the dominant influence of starch gelatinization and expansion dynamics, which are primarily governed by processing temperature and screw speed rather than by minor compositional variations at the ingredient ratio levels tested [69]. Nonetheless, bulk density values in the range of 0.74–1.28 g/mL recorded across formulations are consistent with those reported for dense, chunk-type legume extrudates, distinct from low-density snack puffs where values of 0.10–0.45 g/mL are typical [70].

Water holding capacity was significantly higher in unfermented extruded chunks (264.17–286.24 g/100 g) compared to fermented counterparts (204.46–219.23 g/100 g), despite the observation that fermented pigeon pea flour possessed superior water holding capacity in the raw flour state. This apparent reversal is attributable to the structural modifications induced by extrusion on the fermented flour matrix [71]. Thermomechanical processing disrupts the protein network in fermented flour, which was already partially denatured by fermentation, more extensively than in the native protein matrix of unfermented flour, resulting in a post-extrusion protein structure with reduced capacity for water re-absorption [72]. Rani et al. (2021) reported a similar post-extrusion reduction in water holding capacity for fermented rice-black gram flour, attributing this to the formation of irreversible protein aggregates and the collapse of water-binding hydrophilic domains under high-temperature, high-shear conditions [73]. The practical implication of lower water holding capacity in fermented extrudates is a reduced tendency for product wetness upon rehydration, which may be advantageous from a consumer acceptability perspective.

Oil holding capacity did not differ significantly across formulations (*p* = 0.433), with values ranging from 154.64 to 182.73 g/100 g. This uniformity suggests that the lipid-binding structural attributes of the extrudate matrix, primarily determined by the hydrophobic domains of denatured pigeon pea proteins and the porous starch network, were not substantially altered by the compositional variations tested. These values are comparable to those reported for extruded snacks [68].

Water activity values were significantly lower in fermented–extruded formulations (0.426–0.473) compared to unfermented counterparts (0.456–0.524), with the fermented control recording the lowest value (0.426). Water activity is the primary determinant of microbial stability and shelf life in intermediate-moisture extruded products, and values below 0.60 are generally considered to preclude the growth of most spoilage bacteria and many molds [74]. The reduced water activity observed in fermented extrudates may be attributable to the accumulation of organic acids and soluble carbohydrate fermentation products during the 96 h brine fermentation, which exert a humectant effect and reduces thermodynamic water availability in the extrudate matrix [75]. Other researchers have reported reduced water activity in fermented cereal–legume blends relative to their unfermented equivalents, highlighting the dual contribution of fermentation to both shelf-life extension and compositional modification [76].

The most pronounced differences between fermented and unfermented formulations were observed in hardness and crispness. Fermented extrudates exhibited substantially higher hardness, with the Fermented 90:5:5 formulation reaching 22,802.13 g compared with 5583.12 g in unfermented samples. Crispness followed a similar trend, increasing from 21,130.03 g/s in unfermented formulations to 55,099.48 g/s in the Fermented 90:5:5 treatment. These differences likely result from fermentation-induced modifications to the starch–protein matrix. Beyond partial proteolysis, fermentation reduces phytic acid and other anti-nutritional factors, enhancing protein functionality and promoting stronger protein–starch interactions during extrusion [77]. These changes modify melt rheology, limiting bubble expansion and producing thicker cell walls and a denser cellular structure, which increase fracture resistance and crispness. Fermentation may also promote starch depolymerization and subsequent molecular re-association during cooling, resulting in a more rigid matrix [73]. Similar increases in hardness have been reported in fermented extrudates and fermented–extruded brown rice products, where structural densification reduced expansion and enhanced texture [78]. The superior texture of the Fermented 90:5:5 formulation suggests an optimal starch–protein balance for matrix development [79].

### 4.6. Proximate Composition of Extruded Chunks

The proximate composition of the extruded chunks reflected the combined effects of ingredient formulation and thermomechanical processing on nutrient distribution. Protein content remained statistically non-significant across all extruded formulations (*p* = 0.424), with values ranging narrowly between 18.80% and 19.45%, consistent with the lack of significant protein difference observed in the raw flour ingredients. The preservation of protein content through the extrusion process is a recognized attribute of thermomechanical processing when conducted within controlled temperature ranges; protein denaturation and textural modification occur, but total nitrogen is not substantially lost [80]. Similarly, others have documented no significant reduction in total protein content following extrusion of sorghum–cowpea composite blends, while confirming that while protein digestibility and anti-nutritional factor profiles are altered by extrusion, crude protein content is preserved [81,82]. The consistent protein levels observed across all eight formulations, at approximately 19%, position these extruded chunks as meaningful plant-protein-dense snack products in the context of sub-Saharan African dietary requirements.

Moisture content of the extruded chunks was significantly lower across all formulations (0.61–1.86%) relative to the raw flour ingredients (approximately 11%), as anticipated given the dehydration effect of high-temperature extrusion and the post-extrusion drying conditions [83]. The notably low moisture content of Fermented 90:2.5:7.5 (0.68%) and the Unfermented control (0.61%) is consistent with enhanced moisture evaporation from formulations that exhibited either lower intrinsic water holding capacity or higher pore structure development during expansion [84]. These values fall well within the moisture content range (≤8%) recommended for shelf-stable extruded snack products to prevent microbial spoilage and maintain textural crispness during storage [85].

Crude fat content exhibited significant variation across formulations (*p* < 0.001), with the fermented control recording the highest value (8.42%), followed by the unfermented control (6.05%). The notably higher fat content in control formulations and those with minimal mushroom proportion may be attributed to the concentration effect. In the absence of the high-fiber mushroom fraction, which is inherently very low in lipids (1.90%), the pigeon pea lipid fraction constitutes a proportionally larger share of the extrudate composition. The significant reduction in crude fat content in formulations with higher mushroom inclusion levels reflects the dilution of the pigeon pea lipid pool by the essentially lipid-free mushroom and cassava starch fractions [86]. Additionally, thermomechanical extrusion is known to promote lipid oxidation and volatilization of low-molecular-weight fatty acid fractions, particularly at temperatures above 130 °C, which may further contribute to reductions in extractable fat in high-mushroom-inclusion extrudates [87].

Crude fiber content differed significantly between fermented and unfermented extrudate groups (*p* < 0.001), with unfermented formulations recording consistently higher values (21.11–21.80%) than their fermented counterparts (18.15–19.26%). This difference mirrors the trend observed in the raw flour ingredients, where unfermented pigeon pea exhibited higher crude fiber than its fermented equivalent, and confirms that the fermentation-induced partial solubilization of structural cell wall polysaccharides is not fully reversed or reconstituted during extrusion [55,88]. Consistent with this interpretation, Yağcı et al. (2020) reported lower dietary fiber content in fermented chickpea extrudates compared to unfermented controls, attributing the difference to pre-extrusion enzymatic degradation of cellulosic components by microbial cellulases during fermentation [89]. The high crude fiber content across all formulations (18–22%) is a nutritionally favorable attribute, as dietary fiber intake is consistently associated with reduced risk of cardiovascular disease, type 2 diabetes, and colorectal cancer, and is critically inadequate in the diets of many sub-Saharan African populations [90,91].

Carbohydrate content showed significant variation (*p* < 0.001), with Fermented 90:2.5:7.5 recording the highest value (55.43%) and fermented control recording the lowest (47.00%). As carbohydrates were calculated by difference, the observed pattern reflects the inverse relationships with protein, fat, fiber, ash, and moisture contents across formulations. Notably, formulations with higher cassava starch inclusion showed elevated carbohydrate content, consistent with the dominant carbohydrate fraction of cassava starch (85.75% in the raw ingredient) [92]. This finding highlights the role of cassava starch as the primary modulator of the energy density and glycemic character of the extrudate and indicates that formulation adjustments may be necessary in contexts where low glycemic index products are desirable.

Ash content was significantly higher in mushroom-enriched formulations (4.44–5.22%) compared to cassava starch-dominant formulations, consistent with the high mineral content of mushroom powder documented in the raw ingredient characterization. The elevated ash content across all extruded formulations relative to cassava starch alone confirms the mineral-fortifying contribution of both pigeon pea and mushroom ingredients, and the stability of mineral content through the thermomechanical extrusion process. Minerals were non-volatile and non-susceptible to thermal degradation under the processing conditions employed [93].

## 5. Conclusions

Brine-based spontaneous fermentation of pigeon pea for 96 h at ambient temperature resulted in a consistent decline in pH, confirming lactic acid bacterial activity and demonstrating the feasibility of fermentation as a low-cost pre-processing strategy prior to extrusion. Fermentation significantly influenced extrudate texture, particularly hardness and crispness, likely through partial proteolysis, reduction in anti-nutritional factors, and starch modifications that enhanced protein–starch interactions during extrusion. Protein content remained stable, indicating minimal nitrogen loss during processing, while the low water activity of all products suggests good microbiological shelf stability. The identified optimal extrusion conditions are compatible with single-screw extrusion technology, supporting industrial scalability. Incorporation of button mushroom powder and cassava starch further enhanced ash and dietary fiber contents and improved flavor through umami-active compounds. These findings highlight the potential of combining fermentation and extrusion to develop shelf-stable, nutrient-dense foods from regionally available crops in sub-Saharan Africa and Southeast Asia. However, further studies should evaluate protein digestibility, mineral bioavailability, shelf-life stability, and fermentation microbiota, while employing response surface methodology to optimize processing conditions for large-scale production.

## Figures and Tables

**Table 1:** Functional properties of flours used in production of extruded chunks.

Sample Name	Swelling Power (%)	Solubility (%)	Water Holding Capacity (%)	Oil Holding Capacity (%)	Bulk Density (g/mL)
**Cassava Flour**	278.2 (2.07) ^b^	161.1 (21.9) ^b^	170.65 (4.86) ^a^	177.41 (4.70) ^a^	0.67 (0.001) ^c^
**Fermented Pigeon Pea Flour**	259.8 (23.48) ^ab^	88.8 (0.57) ^a^	272.27 (2.57) ^c^	172.24 (4.61) ^a^	0.63 (0.001) ^b^
**Button Mushroom Powder**	516.4 (0.56) ^c^	77.4 (1.41) ^a^	308.5 (6.87) ^d^	244.69 (1.44) ^b^	0.40 (0.02) ^a^
**Unfermented Pigeon Pea Flour**	228.4 (6.22) ^a^	88 (5.66) ^a^	194.13 (7.16) ^b^	169.05 (0.07) ^a^	0.63 (0.01) ^b^
***p*** **-Value**	<0.001	0.005	<0.001	<0.001	<0.001

Values are mean (SD). Means within a column sharing the same superscript are not significantly different (*p* > 0.05) by post hoc test (Tukey’s HSD).

**Table 2:** Proximate composition of flours used in the production of extruded chunks.

Sample	Protein (g/100 g)	Moisture (g/100 g)	Crude Fat (g/100 g)	Ash (g/100 g)	Crude Fiber (g/100 g)	Carbohydrates (g/100 g)
**Fermented Pigeon Pea Flour**	18.87 (0.15) ^a^	11.05 (0.01) ^a^	7.09 (0.035) ^b^	4.46 (0.16) ^b^	20.82 (0.12) ^a^	37.71 (0.57) ^b^
**Unfermented Pigeon Pea Flour**	19.17 (0.47) ^a^	11.09 (0.07) ^a^	5.70 (0.07) ^a^	4.80 (0.19) ^b^	21.86 (0.27) ^a^	37.38 (0.86) ^b^
**Button Mushroom Flour**	20.71 (0.67) ^a^	10.96 (0.56) ^b^	1.90 (0.31) ^c^	13.11 (0.60) ^a^	20.52 (0.61) ^a^	32.80 (0.00) ^c^
**Cassava Starch**	1.30 (0.25) ^a^	11.09 (0.01) ^a^	0.81 (0.09) ^d^	0.79 (0.29) ^c^	0.26 (0.52) ^b^	85.75 (0.00) ^a^
***p*** **-Value**	0.355	0.002 *	<0.001 *	<0.001 *	<0.001 *	<0.001 *

Values are mean (SD). Means within a column sharing the same superscript are not significantly different (*p* > 0.05) by post hoc test (Tukey’s HSD). * Indicates *p* < 0.05.

**Table 3:** Physicochemical and functional properties of extruded chunks.

Sample Name	Bulk Density (g/mL)	Water Holding Capacity (%)	Oil Holding Capacity (%)	Water Activity	Hardness (g)	Crispness (g/s)
**Fermented 90:7.5:2.5**	1.02 (0.01) ^a^	219.23 (14.60) ^a^	169.83 (3.70) ^a^	0.445 (0.01) ^b^	14,723.81 (2552.46) ^d^	33,512.26 (1080.00) ^f^
**Fermented 90:5:5**	0.91 (0.24) ^a^	207.49 (12.30) ^a^	154.64 (0.25) ^a^	0.443 (0.01) ^b^	22,802.13 (5282.31) ^e^	55,099.48 (292.00) ^g^
**Fermented 90:2.5:7.5**	0.74 (0.11) ^a^	204.46 (5.15) ^a^	176.18 (3.40) ^a^	0.473 (0.14) ^c^	9855.62 (1555.00) ^b^	31,906.35 (358.20) ^e^
**Fermented control**	0.87 (0.02) ^a^	210.33 (1.44) ^a^	165.52 (6.48) ^a^	0.426 (0.00) ^a^	12,614.93 (4583.00) ^c^	12,424.79 (613.03) ^b^
**Unfermented 90:7.5:2.5**	0.84 (0.01) ^a^	280.97 (4.58) ^b^	182.73 (3.25) ^ab^	0.456 (0.01) ^c^	4570.38 (3005.00) ^a^	10,779.43 (240.49) ^b^
**Unfermented 90:5:5**	0.93 (0.06) ^a^	278.14 (4.09) ^b^	166.06 (1.80) ^a^	0.525 (0.01) ^d^	5099.95 (2029.23) ^a^	17,393.92 (1772.71) ^c^
**Unfermented control**	0.76 (0.06) ^a^	286.24 (15.46) ^b^	175.55 (2.06) ^a^	0.474 (0.00) ^c^	5583.12 (809.16) ^a^	21,130.03 (170.00) ^d^
**Unfermented 90:2.5:7.5**	1.28 (0.30) ^a^	264.17 (6.60) ^b^	165.47 (7.83) ^a^	0.477 (0.01) ^c^	4773.92 (220.31) ^a^	2611.81 (1351.06) ^a^
***p*** **-Value**	0.083	<0.001 *	0.433	<0.001 *	0.003 *	<0.001 *

Values are mean (SD). Means within a column sharing the same superscript are not significantly different (*p* > 0.05) by post hoc test (Tukey’s HSD). * Indicates *p* < 0.05, g: grams, g/s: grams per second.

**Table 4:** Proximate composition of extruded chunks.

Sample Name	Moisture Content (%)	Protein (%)	Crude Fat (%)	Crude Fiber (%)	Ash (%)	Carbohydrates (%)
**Fermented 90:7.5:2.5**	1.47 (0.09) ^b^	19.31 (0.14) ^a^	3.62 (0.28) ^b^	19.26 (0.23) ^b^	5.01 (0.20) ^b^	51.36 (0.35) ^b^
**Fermented 90:5:5**	1.42 (0.57) ^b^	19.35 (0.21) ^a^	4.61 (0.28) ^c^	19.19 (0.17) ^b^	5.04 (0.49) ^b^	50.40 (0.67) ^b^
**Fermented 90:2.5:7.5**	0.68 (0.19) ^a^	19.45 (0.35) ^a^	1.15 (0.57) ^a^	18.15 (0.65) ^a^	5.14 (0.25) ^b^	55.43 (0.91) ^c^
**Fermented control**	1.86 (0.46) ^b^	19.25 (0.07) ^a^	8.42 (0.11) ^e^	19.05 (0.55) ^b^	4.44 (0.02) ^a^	47.00 (0.29) ^a^
**Unfermented 90:7.5:2.5**	1.54 (0.36) ^b^	18.82 (0.07) ^a^	1.47 (0.49) ^a^	21.44 (0.23) ^c^	4.68 (0.26) ^a^	52.07 (0.56^) b^
**Unfermented 90:5:5**	1.16 (0.11) ^b^	19.15 (0.07) ^a^	1.32 (0.04) ^a^	21.80 (0.38) ^c^	5.08 (0.36) ^b^	51.49 (0.83) ^b^
**Unfermented control**	0.61 (0.12) ^a^	19.15 (0.21) ^a^	6.05 (0.08) ^d^	21.11 (0.18) ^c^	5.22 (0.40) ^b^	47.86 (0.78) ^a^
**Unfermented 90:2.5:7.5**	1.05 (0.42) ^b^	19.05 (0.35) ^a^	0.73 (0.14) ^a^	21.42 (0.66) ^c^	5.10 (0.11) ^b^	52.65 (0.44) ^b^
***p*** **-Value**	0.0180	0.424	<0.001	<0.001	0.017	<0.001

Values are mean (SD). Means within a column sharing the same superscript are not significantly different (*p* > 0.05) by post hoc test (Tukey’s HSD).

## Data Availability

The original contributions presented in this study are included in the article. Further inquiries can be directed to the corresponding author.

## Abbreviations

The following abbreviations are used in this manuscript:

AW	Water Activity
BD	Bulk Density
°C	Degrees Celsius
cm	Centimeter
g	Gram
g/g	Grams per Gram
g/mL	Grams per Milliliter
g/s	Grams per Second
g/100 g	Grams per 100 Grams
HSD	Honestly Significant Difference (Tukey’s post hoc test)
ICEAP00557	Pigeon Pea Variety Code (ICRISAT breeding line)
ICRISAT	International Crops Research Institute for the Semi-Arid Tropics
kg	Kilogram
L	Liter
L:D	Length to Diameter Ratio
mL	Milliliter
mm	Millimeter
OHC	Oil Holding Capacity
PCA	Principal Component Analysis
pH	Potential of Hydrogen
rpm	Revolutions per Minute
RTE	Ready-to-Eat
SD	Standard Deviation
SP	Swelling Power
*w*/*w*/*w*	Weight to Weight-to-Weight Ratio
WHC	Water Holding Capacity
